# Strengthening New Vaccine Introduction in Low- and Middle-Income Countries: Establishing Hospital-Based Sentinel Surveillance for Vaccine Safety Monitoring

**DOI:** 10.4269/ajtmh.25-0363

**Published:** 2025-09-04

**Authors:** Anna Shaum, Erin Blau, Ashley Longley, Wan-Ting Huang, Jane Gidudu

**Affiliations:** ^1^ Global Immunization Division, Global Health Center, Centers for Disease Control and Prevention, Atlanta, Georgia;; ^2^ Global Health Program, College of Public Health, National Taiwan University, Taipei City, Taiwan;; ^3^ National Taiwan University Children’s Hospital, Taipei City, Taiwan

## Abstract

Enhancing surveillance for adverse events following immunization remains a key global immunization priority. Many low- and middle-income countries (LMICs) are implementing new vaccines without the comprehensive safety monitoring typically conducted in high-income countries. Since 2017, the Global Immunization Safety Team at the US Centers for Disease Control and Prevention, in collaboration with partners, has supported establishing sentinel surveillance systems during vaccine introductions for safety monitoring in LMICs. Through these experiences, many lessons have been learned regarding project initiation, funding opportunities, standardizing data collection, background rate challenges, site selection considerations, and partner coordination. If vaccine safety is prioritized, sentinel surveillance enhances routine monitoring and generates valuable safety data, strengthening the immunization and regulatory programs. As many countries introduce and manufacture vaccines not previously monitored in high-income countries, lessons from safety monitoring during earlier vaccine introductions must be applied. Sustaining the gains in immunization that were hard-earned over the past decades depends on it.

## INTRODUCTION

Enhancing surveillance for adverse events following immunization (AEFI) remains a key priority for sustaining the immunization gains made in the past century.^1^ Many low- and middle-income countries (LMICs) are implementing new vaccines without the comprehensive safety monitoring typically conducted in high-income countries, posing new challenges for advancing global immunization efforts.^2^ New vaccines, such as those against malaria and tuberculosis, will be introduced in the coming years without the necessary vaccine safety systems to conduct robust post-marketing surveillance. This includes systems for identifying adverse events of special interest (AESI), understanding risk factors for specific populations, and developing risk communication plans.^2^^–^^3^

Accelerated vaccine development during emergencies presents additional challenges for monitoring vaccine safety. Over the last decade, numerous public health emergencies have required the rapid mobilization of vaccine safety efforts, including those for Ebola, polio, coronavirus disease 2019 (COVID-19), and mpox. However, these emergencies may generate additional safety data; vaccines may be approved for emergency use, allowing safety data to be monitored in underrepresented but critical target populations (e.g., pregnant and lactating women and immunocompromised individuals).^4^

Over the past 15 years, efforts have been made globally to strengthen passive vaccine safety surveillance, with 43% of WHO countries meeting the new case-based annual reporting indicator in 2022, a threshold of at least 1 serious AEFI reported per 1 million total national or subnational population.^5^ Although passive safety surveillance is a hallmark of an immunization program, systems remain immature in many LMICs. Insufficient funding, a lack of electronic health records, underreporting of AEFI, suboptimal data quality, healthcare worker burnout and turnover, and poor coordination between immunization and regulatory authorities continue to hinder passive surveillance and are now well-documented.^2^^,^^3^^,^^6^ Passive surveillance reports generally cannot be used to determine causal association between AEFIs and vaccination; thus, relying solely on passive surveillance may lead to potential missed safety issues, delayed response to vaccine-related events, and increased hesitancy.^2^ With these continued challenges, enhanced surveillance efforts, such as hospital-based sentinel surveillance, are needed during new vaccine introductions in LMICs, including monitoring for a prioritized and prespecified list of AESI.

### Sentinel surveillance for vaccine safety in LMICs.

Since 2017, the Global Immunization Safety Team at the US Centers for Disease Control and Prevention, in collaboration with partners, has supported the establishment of sentinel surveillance systems for AESI during vaccine introductions. These include systems to monitor the introduction of the typhoid conjugate vaccine (TCV) in India and Zimbabwe (2017–2018),^7^^–^^8^ malaria vaccine in Malawi (2020–2023), fractional dose yellow fever vaccine in Uganda (2018–2022),^9^ novel oral polio vaccine type 2 in Uganda (2022),^10^ and, most recently, the COVID-19 vaccine in Chile, Ethiopia, Malawi, The Gambia, and Uganda (2022–2024; Table 1). Through these experiences, many lessons were learned, and areas were identified for consideration in future work, including project initiation, funding opportunities, standardizing data collection, background rate challenges, site selection considerations, and partner coordination.

**Table 1:** Summary of surveillance projects supported by the Global Immunization Safety Team at the CDC, by year, vaccine, country, and adverse events of special interest, 2017–2024

Year	Vaccine Monitored	Country	AESI*
2017	Typhoid conjugate vaccine	India	Anaphylaxis, encephalitis, generalized convulsions, thrombocytopenia
2018–2019	Typhoid conjugate vaccine	Zimbabwe	Anaphylaxis, aseptic meningitis, generalized convulsions, Guillain-Barre syndrome, non-anaphylaxis hypersensitivity, thrombocytopenia
2018–2022	Fractional yellow fever	Uganda	Anaphylaxis, encephalitis, Guillain-Barre Syndrome, major organ failure, rhabdomyolysis, thrombocytopenia, yellow fever vaccine-associated neurologic disease, yellow fever vaccine-associated viscerotropic disease
2020–2023	Malaria vaccine	Malawi	Abscess, cellulitis, major organ failure, meningoencephalitis, non-anaphylaxis hypersensitivity, Steven-Johnson Syndrome, thrombocytopenia, toxic shock syndrome
2022	nOPV2	Uganda	Acute disseminated encephalomyelitis, acute flaccid paralysis, aseptic meningitis, encephalitis, myelitis, sudden unexplained death
2022–2024	COVID-19	Chile, Ethiopia, Malawi, Uganda	Acute disseminated encephalomyelitis, anaphylaxis, autoimmune hepatitis, Bell’s Palsy, generalized convulsions, Guillain-Barre syndrome, myocarditis, pericarditis, sudden unexplained death, thrombocytopenia, thrombosis or thromboembolism
2022–2024	COVID-19 vaccines in pregnancy	The Gambia	Low birth weight, maternal death, neonatal death, preterm birth, stillbirth

AESI = adverse events of special interest (listed alphabetically); COVID-19 = coronavirus disease 2019; nOPV2 = novel oral polio vaccine type 2.

* Each country developed their own priority list of AESI (e.g., autoimmune hepatitis was only prioritized in Chile for COVID-19). This table reflects what was most often included and identified during surveillance. Full AESI lists are provided in the country-specific publications.

### Project initiation and funding.

**Figure 1.**
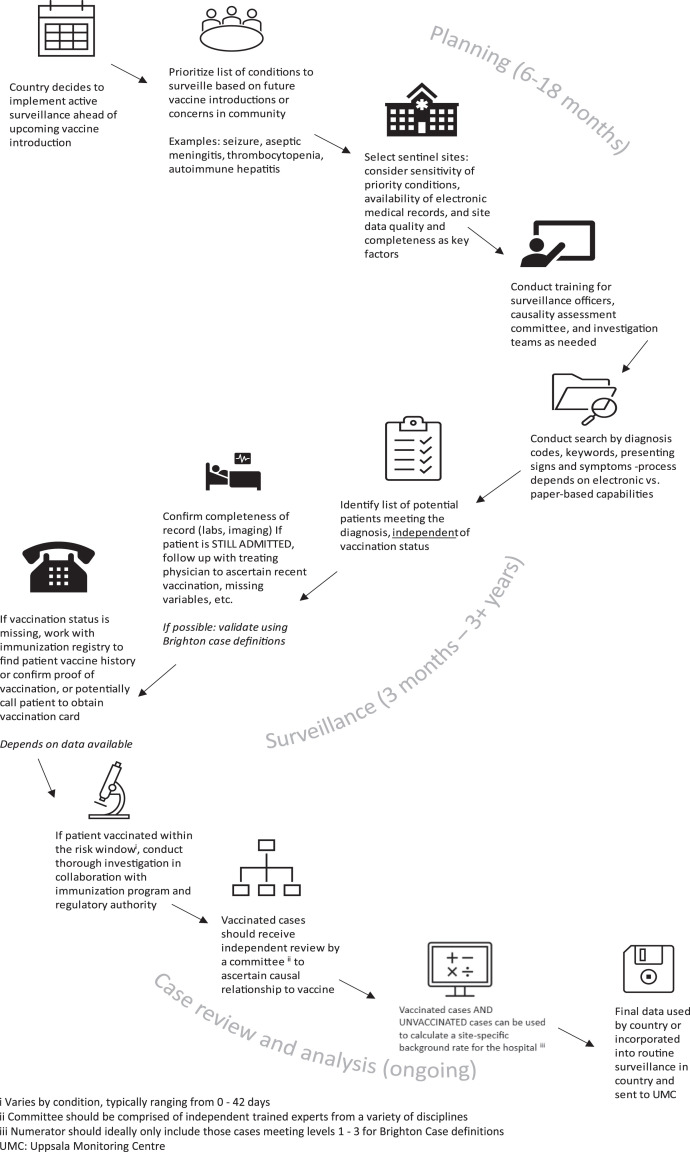
Typical process for implementing prospective hospital-based sentinel surveillance programs in low- and middle-income countries with estimated timelines (US CDC Global Immunization Safety Team).

To begin, the authors collaborated with Ministries of Health to identify funding, select hospital sentinel sites, and generate a list of AESI conditions (mostly serious) based on country priorities and known or theoretical vaccine risks (Figure 1). Implementing enhanced surveillance often requires LMICs to secure additional financial support beyond existing immunization program resources. It has been observed that introducing vaccines may provide opportunities for funding, particularly from external partners such as The Gates Foundation or the Vaccine Alliance (Gavi), which can finance the procurement or implementation of the new vaccine.^7^^–^^8^

Once funding was secured, processes were established for identifying the AESI conditions as part of weekly or daily surveillance at the sentinel hospitals. This often involved a team of dedicated surveillance staff (e.g., physicians, nurses, and surveillance officers) working closely with emergency and inpatient wards in large hospitals to prospectively identify patients with provisional diagnoses indicative of the prespecified AESI list and collect deidentified data on AESI in those patients. For example, in Zimbabwe and Chile, trained nurses supported AESI detection, while in Malawi, a combination of clinicians and surveillance officers led AESI surveillance activities.^8^

### Data availability, collection, and analysis.

The experience of implementing hospital-based sentinel surveillance in LMICs revealed many data quality challenges. Patients are typically referred to these sentinel hospitals from lower-level facilities when their conditions require specialized care, and their medical history, including the onset of illness, may be incomplete. Comprehensive electronic records are often unavailable at the hospitals; in the absence of such records, the manual review of paper-based patient registers and hospital admissions records by trained clinicians and surveillance officers is required to identify potential cases. Moreover, details on vaccinations, including product name, lot number, and vaccination date, are often missing or outdated in the medical records or vaccination cards.^8^^–^^9^

The standardization of safety-related case definitions across hospital-based sentinel surveillance in LMICs is critical to ensure AEFI/AESI comparability. Without standardization, case identification can be challenging to replicate and validate, as was made evident when attempting to identify hypersensitivity non-anaphylaxis cases (a condition lacking a widely accepted standardized case definition) during the introduction of TCV.^8^ The Brighton Collaboration has developed many case definitions for priority vaccine safety conditions that can be used to standardize data collection across settings^11^^–^^12^; these definitions were applied across most of the study hospitals. In Ethiopia, more than 90% of the identified AESI cases met a high number of criteria for Brighton definitions, despite limited diagnostic resources.^11^ In the absence of key case definitions during the COVID-19 pandemic, definitions for conditions that had yet to be defined by Brighton were also generated.^12^

Background rates of conditions under surveillance are needed to interpret findings from hospital-based sentinel surveillance, determining if the observed frequency of an AESI condition exceeds what would be expected in the population in the absence of vaccination. Health authorities can then determine whether the event, such as febrile seizures or thrombocytopenia, is related to the vaccine, rather than another (coincidental) factor, such as malaria or dengue. To generate background rates, denominator data are needed on the catchment population of a hospital sentinel site. Unfortunately, accurate data on catchment populations in LMICs are difficult to estimate, making it challenging to contextualize site-specific findings. In the absence of denominators, hospital-based surveillance provides high-quality safety data that can improve case investigation and causality assessment, which complements the passive surveillance system by generating quality data on serious (hospitalized) AEFI/AESI.^13^ To address the challenges with denominators at tertiary hospitals, countries could consider implementing active surveillance in all health facilities in high-performing districts, as was used during the introduction of the meningococcal serogroup A conjugate vaccine.^14^

### Hospital site selection.

When selecting sentinel hospitals, balancing the sensitivity and specificity of surveillance is critical: secondary or tertiary referral hospitals are more likely to receive patients with serious conditions of interest (e.g., Guillain–Barré syndrome, encephalitis, myocarditis) but may miss some key conditions that are more likely to be identified in outpatient or primary healthcare facilities (e.g., abscess, cellulitis). Furthermore, tertiary care hospitals are often located in large cities and serve as national referral centers, lacking a definable catchment population to calculate background rates. The number of hospital-specific admissions can be used as an imperfect proxy denominator; however, the rates will not be generalizable nationally or regionally. For rare adverse events, hospital-based surveillance may not be adequately powered to conduct observed versus expected analyses for signal detection. In Ethiopia, a readiness assessment was conducted at three candidate hospitals before selecting sentinel sites, aiming to assess indicators such as organizational readiness, the availability of electronic medical records, and data quality.^15^ This example could be used for hospital site selection in similar countries considering active surveillance.

### Partner coordination.

A benefit of sentinel surveillance is the ability to generate timely country-owned vaccine safety data, increasing appreciation for vaccine safety monitoring among healthcare workers. However, because of this increased awareness, hospital-based sentinel surveillance may generate more AEFI data than a country is accustomed to routinely processing, leading to delays in data entry, investigations, and causality assessments. Ensuring that the data management infrastructure is in place before initiating sentinel surveillance is one of the key lessons learned. Sentinel surveillance programs foster much-needed collaboration among national regulatory authorities, national immunization programs, and other key partners. Clear partner communication, coordination, and collaboration are essential in LMICs, where overlapping responsibilities or undefined roles can complicate vaccine safety monitoring. Additionally, funding for new vaccine introductions often emphasizes more healthcare worker training on vaccine safety, resulting in a pool of skilled personnel who can act as “champions” for vaccine safety in the country or region. For example, the hospital-based sentinel surveillance established during the COVID-19 vaccine implementation in Malawi supported more than 2,000 frontline healthcare workers trained on vaccine safety (unpublished report, Malawi, 2024).

### Future directions.

Although gains have been made in strengthening vaccine safety surveillance in LMICs, gaps remain. Comprehensive evaluations of the effectiveness of hospital-based sentinel surveillance, implemented during previous vaccine introductions, are needed, as well as country perspectives on its value in settings where Ministries of Health face competing priorities with limited funding. This includes learning from multi-country active surveillance projects led by other research groups, including the WHO and Gavi, as well as the International Vaccine Institute’s work in Vietnam.^16^^–^^18^ Research is needed to develop the best available background rates of AESI in LMICs, and creative strategies are required to address the challenge of confirming verbally reported vaccine receipt within the risk window of the event.

Estimating the full economic costs of these systems will be beneficial when proposing similar models for future vaccine introductions. Sustainable models for transitioning externally funded programs to Ministries of Health are needed to maintain the efforts, expertise, and established capacity. Collaborating with market authorization holders (MAHs) of the vaccine, as granted by local authorities, is one idea for supporting sustainability because they can be mandated to generate data on their product.^2^ More MAHs may be entering the market as barriers to affordable pharmaceuticals, and frequent zoonotic disease outbreaks have led poorer countries to begin vaccine manufacturing.^19^ In Africa, the Partnership for African Vaccine Manufacturing was created with the aim of pushing for sustainable local manufacturing of 60% of Africa’s vaccine needs by 2040.^20^

As many LMICs introduce and manufacture vaccines not previously monitored in high-income countries, it is essential to continue strengthening vaccine safety surveillance and apply lessons from earlier vaccine introductions. Sustaining the hard-earned gains in immunization depends on it.
